# 16S rDNA-based analysis reveals cosmopolitan occurrence but limited diversity of two cyanobacterial lineages with contrasted patterns of intracellular carbonate mineralization

**DOI:** 10.3389/fmicb.2014.00331

**Published:** 2014-07-08

**Authors:** Marie Ragon, Karim Benzerara, David Moreira, Rosaluz Tavera, Purificación López-García

**Affiliations:** ^1^Institut de Minéralogie, de Physique des Matériaux, et de Cosmochimie, Sorbonne Universités - UPMC Univ Paris 06, CNRS UMR 7590, MNHN, IRD UMR 206Paris, France; ^2^Unité d'Ecologie, Systématique et Evolution, Centre National de la Recherche Scientifique CNRS UMR8079, Université Paris-SudOrsay, France; ^3^Departamento de Ecología y Recursos Naturales, Universidad Nacional Autónoma de MéxicoDF Mexico, Mexico

**Keywords:** *Gloeomargarita*, *Thermosynechococcus*, biomineralization, microbial mat, stromatolite, thermophilic

## Abstract

Cyanobacteria are mainly thought to induce carbonate precipitation extracellularly via their photosynthetic activity combined with the nucleation potential of exopolymeric substances. The discovery in microbialites of the alkaline lake Alchichica (Mexico) of *Candidatus* Gloeomargarita lithophora, a cyanobacterium forming large amounts of intracellular Mg-Ca-Sr-Ba carbonate spherules, showed that intracellular biomineralization in cyanobacteria is also possible. A second cyanobacterium isolated from the same environment, *Candidatus* Synechococcus calcipolaris G9, has been recently shown to also form intracellular calcium carbonates at the cell poles, a capability shared by all cultured species of the *Thermosynechococcus* clade, to which it belongs. To explore the diversity of these two distant cyanobacterial lineages representing two different patterns of intracellular calcification, we designed specific primers against their 16S rRNA genes and looked for their occurrence in a wide variety of samples. We identified the presence of members of the *Gloeomargarita* and *Thermosynechococcus/S. calcipolaris* lineages in microbialites collected from Lake Alchichica and three other neighboring Mexican lakes. The two clades also occurred in karstic areas and in some thermophilic or hypersaline microbial mats collected in South America and/or Southern Europe. Surprisingly, the within-group diversity in the two clades was low, especially within the *S. calcipolaris* clade, with all 16S rRNA gene sequences retrieved sharing more than 97% identity. This suggests that these clades are composed of a limited number of operational taxonomic units (OTUs) with cosmopolitan distribution. Moreover, scanning electron microscopy coupled with energy dispersive x-ray spectrometry showed the presence of intracellularly calcifying *Gloeomargarita*-like cyanobacteria in fresh samples where this clade was relatively abundant, suggesting that these cyanobacteria do precipitate carbonates intracellularly under natural conditions.

## Introduction

The formation of minerals as a consequence of microbial activity is a common phenomenon. Whereas biomineralization seems a tightly controlled process in many unicellular eukaryotes, such as haptophytes (coccolithophorids), radiolarians or diatoms (Bauerlein, 2003), prokaryotes seem to predominantly induce mineral formation indirectly. In this case, mineral precipitation mainly results from the alteration of the cell local chemical environment by its metabolic activities (Newman and Banfield, 2002). A paradigmatic example is carbonate formation.

Until very recently, calcium and/or magnesium carbonates were thought to form essentially extracellularly as a consequence of a variety of bacterial or archaeal metabolisms (Riding, 2000). The oxygenic photosynthesis carried out by cyanobacteria is considered an important driver of carbonate precipitation in cyanobacteria-dominated microbial mats and modern stromatolites (Arp et al., 1999; Reid et al., 2000; Paerl et al., 2001; Dupraz and Visscher, 2005; Dupraz et al., 2009), where carbonates may precipitate in a variety of forms (Gerdes et al., 1994). Similarly, carbonate biomineralization would lead to the phenomenon of “whiting” induced by some planktonic cyanobacteria (Thompson et al., 1997). Two major factors are thought to influence carbonate formation by cyanobacteria. The first is the increase of pH (with the concomitant increase in the concentration of carbonates) resulting from photosynthetic carbon fixation, which affects the saturation index. This “alkalinity engine” leading to local carbonate super-saturation triggers precipitation when Ca^2+^ (or other cations) and when nucleation centers are available in the surroundings. Cation and nucleation center availability thus constitute the second major factor influencing carbonate precipitation and, in cyanobacteria, exopolymeric substances (EPS) that surround cyanobacterial cells are thought to supply them both (Dupraz et al., 2009; Obst et al., 2009). Accordingly, carbonate biomineralization by cyanobacteria has been considered exclusively as an extracellular process. In addition to cyanobacteria, other prokaryotes display metabolic activities susceptible to act as “alkalinity engines,” although they appear to have a lesser impact on a global scale. They include, among others, anoxygenic photosynthetic bacteria (Bundeleva et al., 2012), sulfate reducing bacteria (Visscher et al., 2000; Gallagher et al., 2012), ureolytic bacilli (Hammes et al., 2003) and other Gram positive desert bacteria (Rivadeneyra et al., 1999), myxobacteria (Rodriguez-Navarro et al., 2003) or anaerobic methane oxidizers (Michaelis et al., 2002).

Photosynthesis-induced calcification has been active since early evolutionary times, leaving macroscopic (stromatolite), and other type of traces in the fossil record (Arp et al., 2001; Riding, 2006). This calcification was thought to be extracellular and, in this sense, the use of high-resolution techniques including electron, confocal, and x-ray microscopies has allowed the detection of particular mineral signatures at nanoscale around encrusted cells in modern microbialites (Benzerara et al., 2006) that might be preserved in older fossils. Mineral biosignatures witnessing encrustment are sometimes specifically associated to very particular cyanobacterial species in otherwise highly diversified microbialite communities (Couradeau et al., 2013; Gerard et al., 2013), which confirms previous suspicions of a strong taxonomic control on fossilization/mineralization (Planavsky et al., 2009).

Despite the recognized importance of extracellular calcification by cyanobacteria in modern and past settings, the recent discovery of one cyanobacterium, *Candidatus* Gloeomargarita lithophora, able to produce intracellular carbonates (Couradeau et al., 2012) opened questions about the extent of this phenomenon and its potential contribution to the ancient fossil record (Riding, 2012). The presence of intracellular mineral inclusions in some bacteria has been known for a long time. Granules of elemental sulfur are produced by several anoxygenic phototrophs (Overmann and Garcia-Pichel, 2000) and colorless sulfur bacteria (Robertson and Kuenen, 2006) and many bacteria produce polyphosphate storage inclusions including, notably, cyanobacteria (Seufferheld et al., 2003; Gomez-Garcia et al., 2013), and magnetotactic bacteria (Lins and Farina, 1999). Magnetite (Fe_3_O_4_)-producing bacteria offer the best known example of controlled biomineralization among prokaryotes (Greene and Komeili, 2012; Lefevre and Bazylinski, 2013). However, except for the poorly-studied genus *Achromatium* (*A*. oxalyphera, A. mobile = Macromonas mobilis) (Head et al., 1996), intracellular carbonate inclusions were not known. The discovery of *G. lithophora*, the first-known cyanobacterium producing intracellular carbonates has triggered a series of unexpected findings. Recently, a second cyanobacterium was isolated from the same sample, i.e., biofilms associated to microbialites collected from the alkaline crater lake of Alchichica, Mexico, and maintained in laboratory aquaria (Couradeau et al., 2012). This strain, tentatively named *Candidatus* Synechococcus calcipolaris strain G9 due to its phylogenetic affinity with some strains of the polyphyletic genus *Synechococcus* (Robertson et al., 2001), was found to form carbonate inclusions intracellularly as well. However, *Ca.* S. calcipolaris G9 displayed a remarkable difference with *G. lithophora*, with carbonate inclusions located only at the cell poles, suggesting a potential connection with cell wall division (Benzerara et al., in press).

*Candidatus* Gloeomargarita lithophora and Synechococcus calcipolaris G9 thus represent two distinct mechanisms of carbonate intracellular precipitation occurring in two independent and relatively basal lineages within the Cyanobacteria. How extensive are these biomineralization processes? A partial answer comes from a recent study exploring intracellular carbonate formation across the phylogenetic diversity of cyanobacteria, which shows that this phenomenon has been overlooked in many described cyanobacterial species (Benzerara et al., in press). Also, the exploration of the diversity and abundance of these lineages in various natural environments can lead to a better estimation of their extent and ecological importance. In this work, we aimed at detecting the presence and characterizing the diversity of *G. lithophora* and *S. calcipolaris* lineages in a variety of ecosystems where carbonate precipitation may occur and/or where environmental sequences related to the two cyanobacterial strains had been previously retrieved. Our results show that the two clades are cosmopolitan, although their specific diversity seems reduced, and thrive preferentially in thermophilic microbial mats and calcareous substrates.

## Materials and methods

### Sampling, DNA purification, PCR amplification, cloning, and sequencing

**Figure 1:**
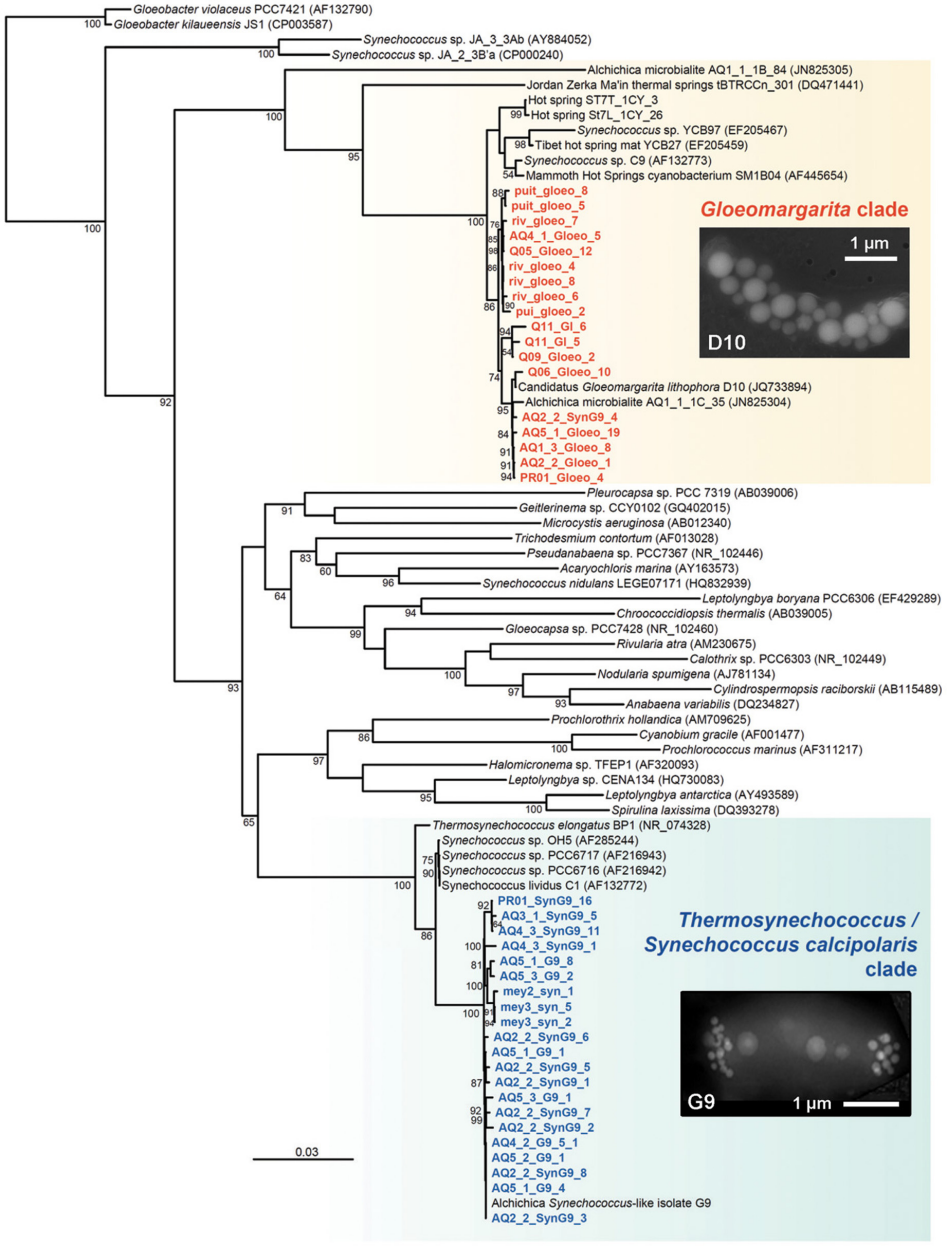
**Phylogenetic tree showing the diversity and the position of two lineages of cyanobacteria producing intracellular carbonate inclusions**. Scanning microscopy photographs of inclusion-bearing cells belonging to representative strains of the *Gloeomargarita* and the *Thermosynechococcus*/*S. calcipolaris* clades are shown as insets. The tree was reconstructed using 1025 conserved positions. The environmental sequences obtained in this work are shown in color. Accession numbers of sequences retrieved from GenBank are given between brackets. Only bootstrap values higher than 50% are given at nodes. The scale bar represents the number of substitutions per a unit branch length.

Samples analyzed in this study (Table 1) were collected during several field trips carried out by the authors in recent years or generously provided by collaborators. Samples collected abroad were fixed in ethanol *in situ*. Samples collected in France were either fixed or transported directly to the laboratory for immediate DNA purification. Prior to DNA extraction, subsamples of ca. 200 μl volume were taken and, if applicable, ethanol was removed after a short centrifugation step (5 min, 10,000 rpm). Samples were then rehydrated in the initial resuspension buffer of the PowerBiofilm DNA isolation kit (MoBio, Carlsbad, CA USA) and the DNA purified using this kit. DNA was eluted in 100 μl of Tris-HCl, pH 8, and conserved at −20°C. Small subunit ribosomal RNA genes were amplified by polymerase chain reaction (PCR) using primers specifically designed to target the *Gloeomargarita* and the *Thermosynechococcus*/*Synechococcus* clades, including some very basal sequences (colored areas in Figure 1). For the former, we designed the primers 69F-Gloeo (5′-AAGTCGAACGGGGKWGCAA) and 1227R-Gloeo (5′-GATCTGAACTGAGACCAAC) and for the latter, primers 209F-synG9 (5′-TGAGGATGAGCTCGCGGTG) and 1231R-synG9 (5′-GAACTGAGCCRYGGTTTAA). PCR reactions were carried out in 25 μl of reaction buffer, containing 1.5 μl of the eluted DNA, 1.5 mM MgCl_2_, dNTPs (10 nmol each), 20 pmol of each primer, and 0.2U Taq platinum DNA polymerase (Invitrogen). PCR reactions were performed under the following conditions: 35 cycles (denaturation at 94°C for 15 s, annealing at 55°C for 30 s, extension at 72°C for 2 min) preceded by 2 min denaturation at 94°C, and followed by 7 min extension at 72°C. Negative (no DNA) and positive (DNA from available targeted cyanobacteria) controls for PCR reactions were used in all cases. DNA from *G. lithophora* and from *S. calcipolaris, T. elongatus*, and *Synechococchus* sp. strains PCC6716 and 6717 was used, respectively, as positive control for the *Gloeomargarita* and the *Thermosynechococcus/S. calcipolaris* clades. In cases where no amplification product was obtained, nested or semi-nested amplifications from amplicons obtained with general cyanobacterial primers CYA106F and CYA-1380R were additionally tested. In general, nested or semi-nested amplification attempts failed, suggesting the absence of the targeted sequences from the corresponding samples. 16S rRNA gene libraries were constructed for all positive amplifications using the TopoTA cloning kit (Invitrogen, Carlsbad, CA, USA) according to the manufacturer's instructions. A total of 35 libraries were constructed for *Themosynechococcus/S. calcipolaris-like* amplicons from different samples and 30 libraries for the *Gloeomargarita* clade. Clone inserts were PCR-amplified using flanking vector primers, and inserts of expected size were partially sequenced (Beckman Coulter Genomics, Takeley, UK) with reverse primer 1227R or 1231R, yielding sequences of ca. 800 bp. A total of 201 cyanobacterial clones were sequenced (33 for *Themosynechococcus/S. calcipolaris* and 168 for *Gloeomargarita* clades). All the sequences obtained were highly similar (>97% identity) but several clones that appeared slightly different were fully sequenced by using forward primers. Complete sequences were assembled using Code Aligner (CodonCode Corporation; www.codoncode.com) prior to phylogenetic analyses. Sequences were deposited in GenBank with accession numbers KJ636536-KJ636761.

**Table 1:** **Samples analyzed for the presence of intracellularly calcifying cyanobacterial clades**.

**Site**	**Sample name**	**Coordinates**	**Collection date**	**Nature of sample**	***Gloeomargarita clade***	***Synechococcus-like G9 clade***
Lake Alchichica, Mexico	AQ1-1	19°25′12.49″N, 97°24′12.35″W (original)	March 2013	Microbialite fragment; maintained in aquarium AQ1 since 2007	+	+
	AQ1-2	19°25′12.49″N, 97°24′12.35″W (original)	March 2013	Microbialite fragment; maintained in aquarium AQ1 since 2007	+	−
	AQ1-3	–	March 2013	Biofilm developing on walls of aquarium AQ1	+	−
	AQ1-4	19°25′12.49″N, 97°24′12.35″W (original)	March 2013	Microbialite fragment collected in 2007 and maintained in aquarium AQ1	+	−
	AQ2-1	19°25′12.49″N, 97°24′12.35″W (original)	March 2013	Microbialite fragment collected in 2007 and maintained in aquarium AQ1	+	+
	AQ2-2	19°25′12.49″N, 97°24′12.35″W (original)	March 2013	Microbialite fragment collected in 2007 and maintained in aquarium AQ1	+	+
	AQ2-3	–	March 2013	Biofilm developing on walls of aquarium AQ2	+	−
	AL2012-Fe	19°25′0.13″N, 97°24′41.07″W	January 2012	Iron-rich microbialite fragment	+	+
	AL2012-1m	19°25′12.49″N, 97°24′12.35″W	January 2012	Microbialite fragment collected at 1 m depth	+	−
	AL2012-5m	19°25′12.49″N, 97°24′12.35″W	January 2012	Microbialite fragment collected at 5 m depth	−	−
	AL2012-10m	19°25′12.49″N, 97°24′12.35″W	January 2012	Microbialite fragment collected at 10 m depth	−	−
	AL2012-15m	19°25′12.49″N, 97°24′12.35″W	January 2012	Microbialite fragment collected at 15 m depth	−	−
	AQ3-1	19°25′12.49″N, 97°24′12.35″W	March 2013	Microbialite collected in 2012 at 15 m depth and kept in aquarium AQ3	−	+
	AQ3-2	19°25′12.49″N, 97°24′12.35″W	March 2013	Microbialite collected in 2012 at 15 m depth and kept in aquarium AQ3	−	−
	AQ3-3	19°25′12.49″N, 97°24′12.35″W (original)	March 2013	Biofilm developing on the water surface of aquarium AQ3	−	−
	AQ3-4	–	March 2013	Biofilm developing on walls of aquarium AQ3	−	−
	AQ4-1	19°25′12.49″N, 97°24′12.35″W (original)	March 2013	Iron-rich, microbialite collected in 2012 and kept in aquarium AQ4	+	−
	AQ4-2	19°25′12.49″N, 97°24′12.35″W (original)	March 2013	Iron-rich, microbialite collected in 2012 and kept in aquarium AQ4	−	+
	AQ4-3	–	March 2013	Biofilm developing on the water surface of aquarium AQ4	−	+
	AQ4-4	–	March 2013	Biofilm developing on the water surface of aquarium AQ4	−	−
Lake La Preciosa, Mexico	PR-01	19°22′31.77″N, 97°23′23.67″W	January 2012	Microbialite fragment	+	+
	PR-02	19°22′31.77″N, 97°23′23.67″W	January 2012	Carbonate crust	−	
Lake Quechulac, Mexico	Q-05	19°22′31.51″N, 97°21′18.14″W	January 2012	Microbialite fragment	+	−
	Q-06	19°22′31.51″N, 97°21′18.14″W	January 2012	Microbialite fragment with Nostoc-like colonies	+	−
	Q-09	19°22′31.51″N, 97°21′18.14″W	January 2012	Microbialite fragment from vertical wall on island	+	−
	Q-10	19°22′31.51″N, 97°21′18.14″W	January 2012	Carbonate crust with endolithic green layer	−	−
	Q-11	19°22′31.51″N, 97°21′18.14″W	January 2012	Small fragments of Q-08	+	−
	AQ5-1	19°22′31.51″N, 97°21′18.14″W (original)	March 2013	Microbialite collected in Quechulac in 2012 and kept in aquarium AQ5	+	+
Lake Atexcac, Mexico	ATX-03	19°20′11.04″N, 97°27′2.01″W	January 2012	Calcifying sediment	+	+
	ATX-04	19°20′11.04″N, 97°27′2.01″W	January 2012	Microbialite fragment	−	−
	AQ5-2	19°20′11.04″N, 97°27′2.01″W (original)	March 2013	Microbialite collected in Atexcac in 2012 and kept in aquarium AQ5	−	+
	AQ5-3	–	March 2013	Biofilm developing on the water surface of aquarium AQ5	−	+
Xcaamal, Yucatan, Mexico	yuc1	20° 36′ 19.4″ N y 89° 42′ 23.2″ W	September 2012	Cenote, plankton	−	−
	yuc3	20° 36′ 19.4″ N y 89° 42′ 23.2″ W	September 2012	Cenote, plankton	−	−
Salar de Llamará, Chile	LLA9-2	21°16′6.61″S, 69°37′5.79″W	March 2012	Anaerobic, hypersaline thermophilic microbial mat	−	−
	LLA9-8	21°16′6.61″S, 69°37′5.79″W	March 2012	Hypersaline microbial mat at redox interface	−	−
	LLA9-16	21°16′6.61″S, 69°37′5.79″W	March 2012	Aerobic, hypersaline microbial mat	−	−
	LLA9-21	21°16′6.61″S, 69°37′5.79″W	March 2012	Aerobic, hypersaline microbial mat	−	−
	LLA11-2	21°16′7.67″S, 69°37′6.45″W	March 2012	Hypersaline microbial mat	−	−
	LLA13-2	21°16′7.17″S, 69°37′5.25″W	March 2012	Hypersaline microbial mat	−	−
Tebinquiche, Salar de Atacama, Chile	TE2-2	23° 8′17.27″S, 68°15′21.94″W	March 2012	Hypersaline microbial mat	−	−
	TE5-2	23° 8′23.41″S, 68°15′58.50″W	March 2012	Crusty, hypersaline microbial mat	−	−
El Tatio hydrothermal field, Chile	TAT1-2	22°20′58.35″S, 68° 0′31.84″W	March 2012	Thermophilic (~45°C) microbial mat	−	+
	TAT4-2	22°20′58.35″S, 68° 0′31.84″W	March 2012	Thermophilic (~45°C) microbial mat	−	−
Geyser Blanco, El Tatio hydrothermal field, Chile	GB2-2	22°21′23.85″S, 68° 1′22.58″W	March 2012	Thermophilic (~50°C) microbial mat	−	−
	GB3-2	idem	March 2012	Thermophilic (~70°C) microbial mat	−	−
Salada de Peine, Salar de Atacama, Chile	SP2-2	23°40′28.01″S, 68° 8′49.59″W	March 2012	Hypersaline calcifying microbial mat	−	−
	SP3-2	23°40′27.23″S, 68° 8′48.83″W	March 2012	Hypersaline calcifying microbial mat	−	−
Joute River valley, Parc de Grands Causses, France	Val-Jou	44°12′00″N, 3°23′00″E	June 2013	Microbial mat on rock	−	−
St Marcelin, River Tarn, Parc de Grands Causses, France	Riv-Tarn	44°13′17″N, 3°13′22″E	June 2013	Microbial mat on rock	+	−
Meyrueis, Parc de Grands Causses, France	Meyrueis1	44°11′06″N, 3°26′18″E	June 2013	Microbial mat on rock	−	−
	Meyrueis2	idem	June 2013	Microbial mat on rock	−	+
	Meyrueis3	idem	June 2013	Microbial mat on rock	−	+
Lavogne de Villeneuve, France	Puit-Lavo	44°15′19″N, 3°25′59″E	June 2013	Microbial mat	+	−
Lavogne de Villeneuve, France	Lavo	44°14′21″N, 3°32′08″E	June 2013	Microbial mat	−	−
Lavogne Hure, France	Lavo 0,2	44°15′19″N, 3°25′59″E	June 2013	Plankton 0,2–5 μm	−	−
Lavogne Hure, France	Lavo 5	44°15′19″N, 3°25′59″E	June 2013	Plankton > 5 μm	−	−
Lake Pavin, Auvergne, France	Pav	45°29′44.81″N, 2°53′17.24″E	June 2013	Plankton > 0,2 μm	−	−
Fuente de los Baños, Parc National de Ordesa, Spain	FB	42°30′57.70″N, 0°6′16.98″E	September 2013	Microbial biofilm at carbonate spring	−	−
Salada de Chiprana, Spain	Chip	41°14′31.13″N, 0°10′57.94″W	March 2013	Hypersaline microbial mat	+	−
Mayotte, Indian Ocean, France	MAY1-2013	12°35′7.55″S, 44°58′39.13″E	April 2013	Microbialite biofilm	−	−
	MAY2-2013	12°35′7.55″S, 44°58′39.13″E	April 2013	Hydrothermal chimney	−	−

When not indicated otherwise, microbialite samples were collected near the water surface.

### Phylogenetic analyses

Environmental 16S rRNA gene sequences retrieved from our samples were compared with sequences in the GenBank database (http://www.ncbi.nlm.nih.gov/) by BLAST (Altschul et al., 1997). We retrieved the closest sequences found in the database and included them in an alignment containing also sequences from the closest cultivated members and some representative sequences of major cyanobacterial taxa. Sequences were aligned using MUSCLE (Edgar, 2004). Ambiguously aligned positions and gaps were eliminated using Gblocks (Castresana, 2000). The resulting sequence alignments were used as input to build phylogenetic trees by approximate maximum likelihood using Fasttree (Price et al., 2010) with a General Time Reversible (GTR) model of sequence evolution, and taking among-site rate variation into account by using a four-category discrete approximation of a Γ distribution. ML bootstrap proportions were inferred using 1000 replicates. Trees were visualized with FigTree (http://tree.bio.ed.ac.uk/software/figtree/).

### Scanning electron microscopy and energy dispersive x-ray spectrometry

Samples of freshly collected aquaria biofilms and microbial mat samples were briefly rinsed in abundant distilled water to avoid the formation of extracellular precipitates upon drying, as previously described (Couradeau et al., 2012, 2013). Then, small sample fragments were deposited onto on formvar-coated transmission electron microscopy (TEM) cupper microscopy grids and let dry. Scanning electron microscopy (SEM) analyses were performed using a Zeiss ultra 55 SEM equipped with a field emission gun. Images were collected in backscattered electron (BSE) mode with a Zeiss Ultra 55 FEG-SEM operating at 10 kV with a 30 μm aperture and a working distance of 7.5 mm using the Angle selective Backscattered (AsB) detector. This mode of imaging provides a contrast which is sensitive to the average atomic number, hence allowing detecting intracellular carbonate inclusions. Elemental compositions of the observed mineral precipitates were directly determined by energy dispersive x-ray spectrometry (EDXS) using an EDS QUANTAX detector and the software ESPRIT (Bruker Corporation, Germany) as previously achieved by Couradeau et al. (2013).

## Results and discussion

### Detection of *gloeomargarita* and *thermosynechococcus/s. calcipolaris* clades in environmental samples

The first step to detect the environmental presence of the two groups of cyanobacteria to which *Candidatus* G. lithophora and S. calcipolaris G9 belong involved the design specific primers to amplify their respective 16S rRNA genes. These primers targeted a relatively broad diversity of cultured and uncultured cyanobacteria forming a monophyletic cluster with each of them (colored areas in Figure 1), and were validated with control DNA from available cultured species (see Materials and Methods). In the case of *Gloeomargarita*, our primers were designed to capture the diversity within a large cluster including the only cultivated member of the group, *Candidatus* G. lithophora, as well as several close environmental sequences, mainly retrieved from thermophilic mats or hot springs, but also from more distant cyanobacteria, including the early-branching operational taxonomic unit (OTU) AQ1-1-1B-84 from Lake Alchichica microbialites (Couradeau et al., 2011) and a sequence from a filamentous thermophilic cyanobacterium (tBTRCCn 301) retrieved from a thermal spring in Jordan (Figure 1). In the case of *Ca.* S. calcipolaris G9, the clade seemed less diverse, with relatively few related sequences in GenBank. Indeed, the closest sequences to *Ca.* S. calcipolaris G9 corresponded to those of cultured species, including four highly related *Synechococcus* species (*S. lividus* and *Synechococcus* sp. PCC6716, PCC6717, and OH5) and *Thermosynechococcus elongatus*, all of which are thermophilic (Dyer and Gafford, 1961; Miller and Castenholz, 2000; Nakamura et al., 2002) (Figure 1). Therefore, in the following, we will refer to this clade indistinctly as the *Thermosynechococcus* clade or the *Synechococcus calcipolaris*-like clade.

Because both *G. lithophora* and *S. calcipolaris* G9 were enriched from carbonate microbialites of the alkaline Lake Alchichica and because their closest relatives in GenBank had been most often identified in thermophilic microbial mats, we searched to identify these clades in a broad collection of samples collected worldwide that included thermophilic settings compatible with photosynthesis (40–70°C) and carbonate-rich environments, such as microbialites and karstic areas (Table 1). In particular, we explored the presence of the two clades in different microbialite samples from Lake Alchichica collected at different locations along the shore of the lake, time, and depths (from 0 to 15 m depth). We also analyzed microbialite samples from neighboring lakes, including Quechulac, La Preciosa, and Atexcac. We tried to amplify the 16S rRNA genes from the two clades in a total of 62 samples (Table 1). From these, amplicons of the expected size were obtained in a total of 20 samples for the *Gloeomargarita* clade, whereas only 15 were obtained for the *S. calcipolaris*-like clade. All of them were cloned and, in the vast majority of cases, the corresponding clone sequences belonged to the targeted groups (see below), showing a high specificity of the designed primers. Only in a few instances, always after nested or semi-nested PCR, were other cyanobacterial sequences retrieved.

The *Gloeomargarita* group was detected in microbialites from Alchichica, Quechulac, La Preciosa, and Atexcac lakes (Table 1). However, *Gloeomargarita* identification consistently failed in deep microbialites, being undetected in Alchichica samples from 5, 10, and 15 m as well as in microbialites collected at 15 m depth and kept afterwards in laboratory aquaria. By contrast, *Gloeomargarita* 16S rRNA genes were amplified from all Alchichica microbialites collected down to 1 m depth, including those maintained in aquaria. They were also amplified from Atexcac, La Preciosa, and Quechulac, but not from all the samples tested (Table 1). Heterogeneous amplification from different samples coming from the same lake was a much stronger trend for the *S. calcipolaris*-like group. Indeed, its detection in microbialites from Alchichica and surrounding lakes was patchy. This may simply reflect the highly heterogeneous nature of the microbialite environment, which may result in local differences in the distribution of different microbial taxa. However, given that this should also affect the amplification of *Gloeomargarita* 16S rDNAs, this patchier detection suggests that, although similarly largely distributed in these lacustrine microbialites, the *Synechococcus*-like G9 clade is less abundant as compared to *Gloeomargarita*.

In addition to the microbialites in the Alchichica area, the *Gloeomargarita* clade was also detected in microbial mat samples collected from karstic environments in the Southwest of France (Parc Naturel Régional des Grands Causses and surrounding areas) as well as in a hypersaline microbial mat of the Salada de Chiprana, Spain (Table 1). Interestingly, carbonate precipitation occurs within Chiprana hypersaline mats (Vidondo et al., 1993), most likely due to a combination of photosynthetic and sulfate-reducing activities (Ludwig et al., 2005). Similarly, the *S. calcipolaris*-like group was detected in mats growing on carbonate rocks from karstic areas in Southern France but also in a microbial mat from El Tatio, a vast hydrothermal field located at high altitude (4300 m) in the Chilean Andes (Table 1). Notably, neither group was detected in plankton samples from karstic systems (encompassing a Mexican cenote and a sample from Southern France) or from the French crater lake Pavin.

Although more prevalent in crater lake microbialites, the identification of the *Gloeomargarita* and the *S. calcipolaris*-like groups in samples coming from other, very distant locations, indicates that the distribution of these lineages is cosmopolitan. Ecologically, the two cyanobacterial groups seem to thrive preferentially in thermophilic mats (Dyer and Gafford, 1961; Miller and Castenholz, 2000; Nakamura et al., 2002; Couradeau et al., 2012) or in carbonate rocks, either lake microbialites or carbonates from karstic areas. Interestingly, other deep-branching cyanobacterial groups, such as the *Gloeobacter* lineage, are also associated with rock environments (Mares et al., 2013).

### Within-group diversity of *gloeomargarita* and *thermosynechococcus/s. calcipolaris* lineages

Surprisingly, even if our specific primers were designed to capture a wide phylogenetic diversity, and even if the sequences obtained came from very different locations, all the 16S rRNA gene sequences that we obtained were all very similar sharing, within each clade, more than 97% identity, the cut-off usually established to define prokaryotic OTUs (Figure 1 and Figure S1). Despite this high similarity, there were some differences possibly reflecting strain variation. Some of this strain variation might reflect local adaptations influenced by geographic and/or physico-chemical differences, both types of factors being very difficult to disentangle. This might perhaps be the case of *S. calcipolaris* G9-like sequences from Atexcac or Meyrueis/El Tatio or of some *Gloeomargarita*-like sequences from Chiprana, Alchichica or Quechulac (Figure S1). To really check for strain differentiation, studies using additional, more variable, phylogenetic markers (e.g., multi-locus sequence typing) would be needed.

In summary, the diversity of *Gloeomargarita* and the *S. calcipolaris* G9-like group was extremely limited within samples and also across continents and ecosystems. Their low intra-group diversity and the fact that these cyanobacteria (especially the *Thermosynechococcus* group) did not appear dominant in many of the analyzed samples suggest that they are specialists. These two cyanobacterial lineages provide good examples of the tenet “everything is everywhere, but the environment selects” (Baas-Becking, 1934; Martiny et al., 2006), since they encompass a few cosmopolitan OTUs subjected to strong environmental selection.

### Calcification potential in the environment

Among prokaryotes, it is well known that similar 16S rRNA sequences do not necessarily imply similar phenotypic properties. Therefore, even if we detected 16S rRNA gene sequences very closely related to the two Alchichica intracellularly calcifying cyanobacteria *G. lithophora* and *S. calcipolaris* G9, their capability to form intracellular carbonates remained to be shown. Therefore, we carried out scanning electron microscope (SEM) observations on fresh environmental samples where they might be relatively abundant to look for the presence of intracellularly calcifying cyanobacterial cells.

**Figure 2:**
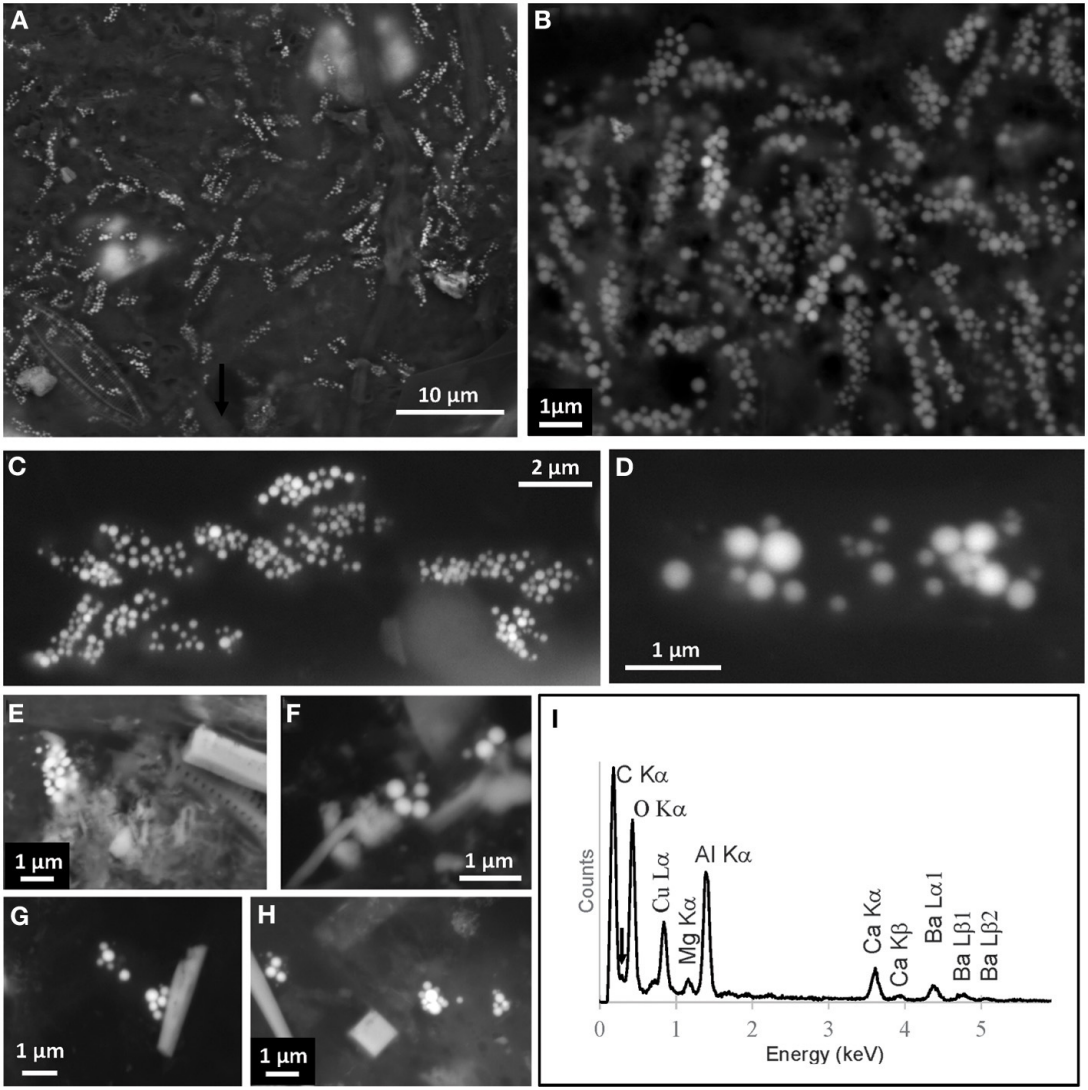
**Scanning electron microscopy images of cyanobacteria with intracellular carbonate inclusions in environmental samples. (A,B)** Images of *Gloeomargarita*-like cells growing in the biofilm of aquarium AQ1 where *G. lithophora* was isolated from. **(C)** *Gloeomargarita*-like cells in a biofilm sample from aquarium AQ2. **(D–H)** Individual *Gloeomargarita*-like cells in natural thermophilic mat samples (Algerian hot spring St7, Meskoutine; Amarouche-Yala et al., under review). Cell boundaries are difficult to observe due to the absence of fixation and staining to prevent carbonate dissolution. **(I)** Energy dispersive x-ray spectrometry (EDXS) analysis on intracellular inclusions of the cell shown in **(D)** from the hot spring St7 microbial mat. C, N (arrow), O, Ca, and Ba can be detected from the inclusions in the cells. Al and Cu emission lines are due to the TEM copper grid and the aluminum sample holder.

The *Gloeomargarita* clade is more diverse, but so far only *G. lithophora* has been shown to possess intracellular carbonate precipitates both in enrichment cultures and in the biofilm it was originally enriched from Couradeau et al. (2012). Sequences affiliating to *G. lithophora* were also present in Alchichica microbialites, but their abundance in such a complex and diversity-rich environment seemed too low for SEM detection to be feasible (Couradeau et al., 2011). By contrast, confirming original observations, our analysis of wall-biofilm samples from two different aquaria (AQ1 and AQ2) showed a high density of *G. lithophora* cells containing a huge amount of intracellular Ca, Mg, Sr, and Ba-enriched carbonate precipitates (Figures 2A–C), as confirmed by energy dispersive x-ray spectrometry (EDX) analyses (Couradeau et al., 2012, and data not shown). This remarkable abundance suggests that *G. lithophora* is particularly fit to this type of biofilm environment and/or that is among the first biofilm-forming organisms on solid substrates (the glass wall in this case). *G. lithophora* not only forms carbonate inclusions in this semi-natural environment, but seems to have a much higher precipitate content than cultures in BG11 medium (Benzerara et al., in press).

In addition, a recent study of cyanobacterial diversity showed a remarkable relative abundance of sequences (up to 12%) belonging to the *Gloeomargarita* clade in a calcifying microbial mat from a hot spring at Meskoutine, Algeria (site St7, 55–70°C) (Amarouche-Yala et al., under review). We therefore looked for the presence of cells containing intracellular carbonates using SEM. As can be seen in Figures 2D–H, we identified a number of cells compatible in size and shape with *Gloeomargarita* sp. and containing intracellular biominerals. EDX analyses on these precipitates showed that they contained carbon, calcium and barium, and lacked phosphorous or sulfur (Figure 2I). This suggests that these inclusions are carbonates of calcium and barium. Barium occurrence in the relatively divergent St7 *Gloeomargarita*-like cells together with previous observations on *G. lithophora* (Couradeau et al., 2012) strongly argue for the general capacity of this clade to concentrate barium intracellularly. Our observations suggest that members of the apical *Gloeomargarita* clade, encompassing *G. lithophora* and the St7 phylotypes (Figure 1) do form intracellular carbonates in their natural environment. However, whether more basal OTUs in the clade can produce intracellular carbonate inclusions or not remains to be determined.

In the case of the *Thermosynechococcus*/*S. calcipolaris* G9 group, all the cultured species examined, including the more basal *T. elongatus*, do form intracellular calcium carbonates in culture (Benzerara et al., in press). However, we have never been able to observe individual cells with their characteristic polar carbonates in environmental samples. Given the universal ability of members of this clade to precipitate carbonate inclusions intracellularly in culture media, and given that *Gloeomargarita* cells form large amounts of carbonate inclusions in the natural environments where they are found, it is likely that *S. calcipolaris* G9-like cells do also produce intracellular precipitates in natural environments. The failure to identify them in natural samples may be related to their low relative abundance in the environments examined. Whether members of this cosmopolitan group are more abundant in other, yet-to-explore ecosystems remains to be determined.

In summary, our study shows that two relatively basal lineages of cyanobacteria producing intracellular carbonates by two different mechanisms are cosmopolitan and exhibit preference for moderately thermophilic microbial mats and microbialites or other, karstic carbonate-rocks. Some of these cyanobacteria, at least members of the *Gloeomargarita* clade, can be relatively abundant locally. Therefore, their contribution to carbonate precipitation in those ecosystems on the long run may be far from negligible. Furthermore, since these lineages are relatively early-branching (Criscuolo and Gribaldo, 2011; Couradeau et al., 2012; Shih et al., 2013), if their ability to produce intracellular carbonates was ancestral, they may have been important contributors to carbonate production in the geological record (Riding, 2000), especially because thermophilic mats and microbialites were likely more prevalent in the past. The idea that intracellular calcification may have been more important in the past is further reinforced by the recent discovery that this phenomenon is much more widespread than previously thought among cyanobacteria (Benzerara et al., in press). Altogether, these observations lend credit to the hypothesis that extracellular fossilization in cyanobacteria appeared relatively late, perhaps linked to the development of carbon concentrating mechanisms by cyanobacteria, major environmental changes at the surface of the Earth and/or the appearance of conspicuous EPS layers, and might help to explain why cyanobacterial microfossils are not detectable in very old fossil stromatolites (the so-called “Precambrian enigma”) (Couradeau et al., 2012; Riding, 2012).

### Conflict of interest statement

The authors declare that the research was conducted in the absence of any commercial or financial relationships that could be construed as a potential conflict of interest.
