# Supplementary material for: 16S rDNA-based analysis reveals cosmopolitan occurrence but limited diversity of two cyanobacterial lineages with contrasted patterns of intracellular carbonate mineralization
Source: Front Microbiol. 2014 Jul 8;5:331. doi: 10.3389/fmicb.2014.00331 (PMC4085569; doi:10.3389/fmicb.2014.00331)
Supplement: Supplementary file 1 [file Presentation1.PDF]

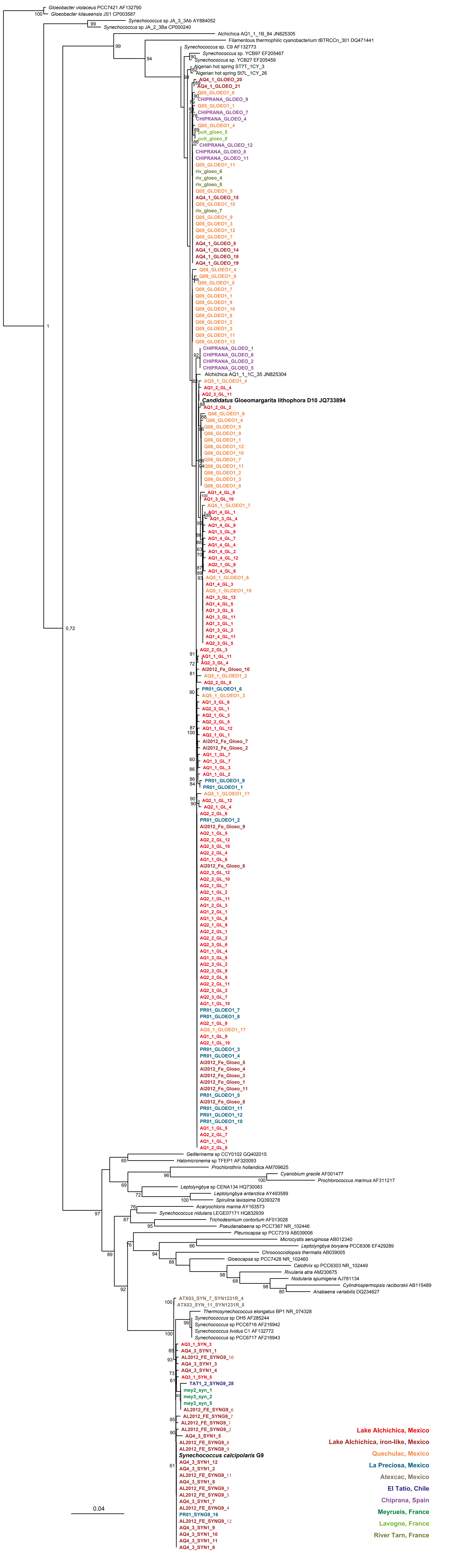

FIGURE S1. Phylogenetic tree showing the diversity of the Gloeomargarita and the Synechococcus-like G9 clades of cyanobacteria identified in various environmental samples. The tree was reconstructed using 688 conserved positions to be able to incorporate all partial sequences analyzed (ca. 200 environmental sequences). The environmental sequences obtained in this work are shown in colors according to their original habitat. Accession numbers of sequences retrieved from GenBank are given for reference sequences. Only bootstrap values higher than 50% are given at nodes. The scale bar represents the number of substitutions per a unit branch length.
